# QUANT: a long-term multi-city commercial air sensor dataset for performance evaluation

**DOI:** 10.1038/s41597-024-03767-2

**Published:** 2024-08-21

**Authors:** Sebastian Diez, Stuart Lacy, Josefina Urquiza, Pete Edwards

**Affiliations:** 1 Centro de Investigación en Tecnologías para la Sociedad, Universidad del Desarrollo, Santiago, CP 7550000 Chile; 2 Wolfson Atmospheric Chemistry Laboratories, University of York, York, YO10 5DD UK; 3 Grupo de Estudios de la Atmósfera y el Ambiente (GEAA), Universidad Tecnológica Nacional, Facultad Regional Mendoza (UTN-FRM), Cnel. Rodriguez 273, Mendoza, 5501 Argentina; 4 Consejo Nacional de Investigaciones Científicas y Técnicas (CONICET), Buenos Aires, Argentina

**Keywords:** Environmental monitoring, Atmospheric chemistry

## Abstract

The QUANT study represents the most extensive open-access evaluation of commercial air quality sensor systems to date. This comprehensive study assessed 49 systems from 14 manufacturers across three urban sites in the UK over a three-year period. The resulting open-access dataset captures high time-resolution measurements of a variety of gasses (NO, NO_2_, O_3_, CO, CO_2_), particulate matter (PM_1_, PM_2.5_, PM_10_), and key meteorological parameters (humidity, temperature, atmospheric pressure). The quality and scope of the dataset is enhanced by reference monitors’ data and calibrated products from sensor manufacturers across the three sites. This publicly accessible dataset serves as a robust and transparent resource that details the methods used for data collection and procedures to ensure dataset integrity. It provides a valuable tool for a wide range of stakeholders to analyze the performance of air quality sensors in real-world settings. Policymakers can leverage this data to refine sensor deployment guidelines and develop standardized protocols, while manufacturers can utilize it as a benchmark for technological innovation and product certification. Moreover, the dataset has supported the development of a UK code of practice, and the certification of one of the participating companies, underscoring the dataset’s utility and reliability.

## Background & Summary

In a world where the impacts of air pollution are increasingly relevant^1^, sensor technologies emerge as potentially transformative tools^2^ designed to augment monitoring^3^ and intervention strategies^4^. While the advantages of extensive spatial coverage^5^ and real-time data collection^6^ are compelling, the accuracy^7^ and reliability^8^ of the data obtained from air sensors remain fundamental concerns^9^. End-users must have a clear and accurate understanding of the performance of sensors in real-world environments to make well-informed decisions^10^. This is particularly critical in the realm of commercial applications, where proprietary systems often operate as “black boxes”^11^ providing users with limited insight into data processing mechanisms.

Despite the rapid evolution of commercial systems, significant challenges remain, such as cross sensitivities^12^, internal consistency^13^, signal drift^14^, long-term performance^15^, data coverage^16^, and environmental influences^17^. The wide range of devices available on the market and few impartial real-world evaluations make it hard for end-users to predict device performance in specific applications. Furthermore, the variety of assessment methodologies^18^, the use of diverse data quality metrics, and the lack of robust open-access datasets render the comparison of studies a complex task.

Recent studies have addressed sensor performance evaluations with various approaches, albeit with some limitations. For example, Park *et al*.^19^ evaluated 30 nodes in urban settings (measuring CO, NO_2_, O_3_, PM_2.5_, and PM_10_) and conducting short-term evaluations. Jiao *et al*.^20^ focused on a suburban environment (evaluating sensors for NO_2_, O_3_, PM_2.5_, and SO_2_) over eight months. Collier-Oxandale *et al*.^21^ conducted laboratory and field tests in California with 28 gas sensors (for CO, NO_2_, and O_3_), while Liu *et al*.^7^ extended the duration of the study to 13 months in Australia (evaluating PM_2.5_ and CO) with an unspecified number of sensors. Munir *et al*.^6^, the only evaluation in the UK (Sheffield) found in the literature, focused on the evaluation of 10 sensors (measuring NO, NO_2_, and CO) over a year. None of the mentioned studies seem to provide public data access.

The QUANT dataset represents, to the best of our knowledge, the most extensive open-access evaluation of commercial sensor systems on a global scale to date^18^. Part of the UK Research and Innovation Clean Air programme, the QUANT (Quantification of Utility of Atmospheric Network Technologies) project, aims to tackle these issues by evaluating the performance of commercial sensor systems within urban environments across the UK^18^. Moreover, limited access to highly accurate measurement instruments —and the expertise required to effectively employ them— continues to restrict improvements in these newer technologies^22^. To address this issue, collaborative efforts are needed to transform academic knowledge into practical insights that can benefit the wider user community.

**Fig. 1:**
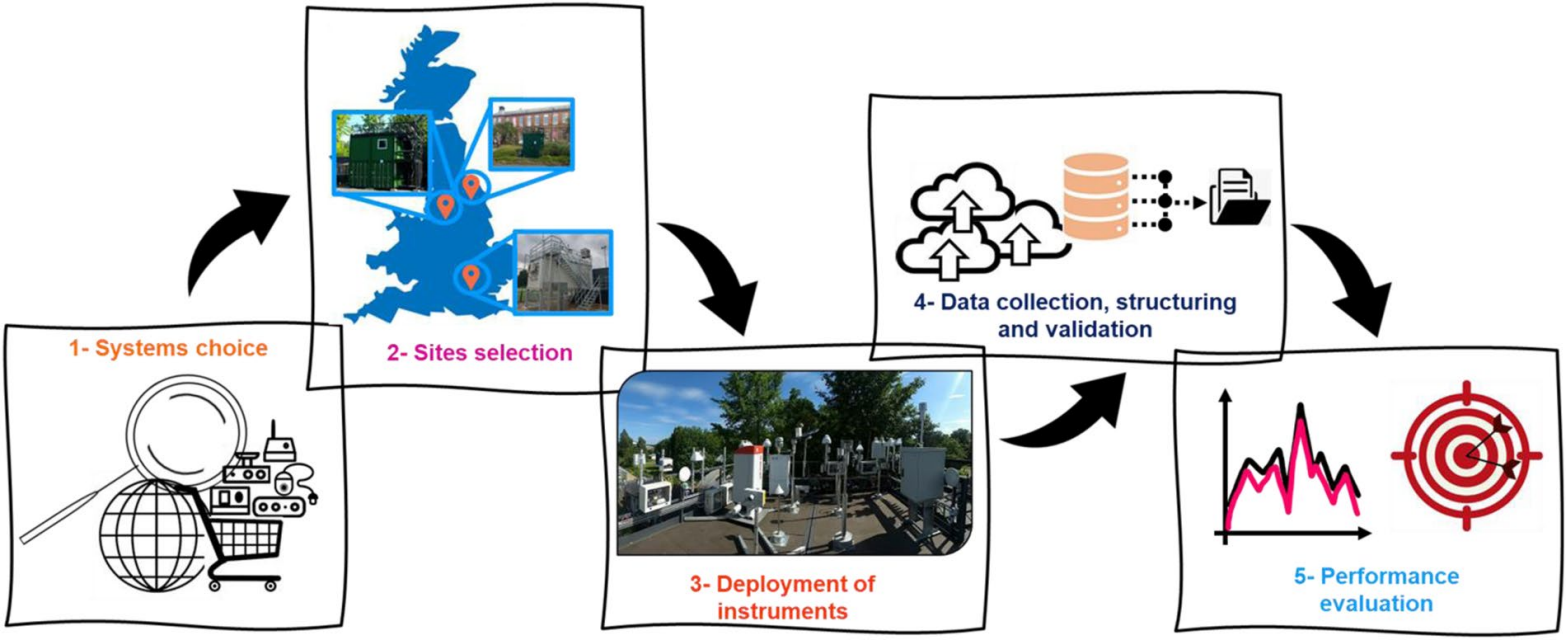
Main QUANT study workflow description: first, market research was conducted to identify and select suitable sensor systems; second, three urban sites in the UK were chosen for implementation; third, commercial devices were installed in the selected locations; fourth, the collection, organization and preliminary validation of the data was carried out; fifth, performance assessment (not part of this paper). For the WPS, steps 1 and 2 differed slightly, as participation was voluntary (no selection on our part) and took place at a single site (MAQS). See the methods section for more details.

Through the QUANT project, a wide array of sensor technologies was systematically deployed across three representative urban sites in the UK, divided into two distinct phases: the first called “Main QUANT” and the second, the “Wider Participation Study” (WPS). The chosen sites for this initiative included two urban-background measurement supersites: the Manchester Air Quality Supersite (MAQS) and the London Air Quality Supersite (LAQS), along with a roadside monitoring station in York (YoFi), part of the Automatic Urban and Rural Network (AURN). The workflow of the QUANT study is depicted in Fig. 1.

The Main QUANT phase, spanning nearly three years (December 2019 to October 2022), focused on a long-term, transparent evaluation of selected commercial sensor devices, acquiring and assessing 26 units from 5 commercial brands at the MAQS, LAQS, and YoFi sites. Additionally, with the aim of fostering sensor innovation, the WPS was organized, covering the period from June 2021 to October 2022, and conducted entirely at the MAQS site. This second stage offered a cost-free opportunity for any commercial entities to engage in an impartial evaluation. During the WPS, 23 units from 9 companies were assessed. Altogether, 49 commercial devices were evaluated, yielding 119 gas (including NO, NO_2_, O_3_, CO and CO_2_), 118 particulate matter (PM) measurements (almost all measuring PM_1_, PM_2.5_, PM_10_), and a number of meteorological measurements (including temperature (Temp), relative humidity (RH), and atmospheric pressure (Pres)). Throughout both phases, the study encompassed a range of meteorological conditions and pollutant concentrations, providing a comprehensive view of sensor performance in varied environments. In order to minimize uncertainties external to the systems and the companies involved, the study implemented a robust study design complemented by stringent quality control procedures. To rigorously evaluate system performance and identify some of their strengths and weaknesses, comprehensive reference (and equivalent-to-reference as defined by the European Commission^23^) measurement data were collected throughout the study. Making use of this extensive reference data, the study also explored how local corrections by manufacturers influenced sensor performance, further enriching the understanding of each device’s capabilities.

The QUANT dataset empower stakeholders—including researchers, policymakers, and urban planners—to understand the behavior of commercial sensor technologies in various environments and refine correction models for specific applications. Policymakers, for instance, can leverage this data to refine sensor deployment guidelines and develop standardized protocols. The insights from this dataset have already contributed to the development of the UK PAS 4023:2023 (https://standardsdevelopment.bsigroup.com/projects/2022-00710), which outlines best practices for the selection, deployment, and quality assurance of air quality sensor systems. For companies not participating in the original study, the dataset can reveal challenges associated with long-term sensor evaluations and pinpoint specific opportunities for innovation. For participating companies, on the other hand, it serves as an invaluable resource for benchmarking products and facilitating improvements, as well as for certification processes. An example is AQMesh, which recently leveraged the QUANT data to achieve UK MCERTS certification (see https://www.csagroup.org/wp-content/uploads/MC240422.pdf).

## Methods

### Systems selection

The selection and purchasing process of the devices for the Main QUANT took place between September and October 2019. Our choice of sensor systems was informed by specific criteria:

- Measure key pollutants: each device had to measure either NO_2_ or PM_2.5_, due to their importance in the UK regulatory framework, and we also opted to include devices that also reported O_3_ due to its importance globally.
- High temporal resolution: the sensors were required to provide data at resolutions ranging from 1 to 15 minutes, to allow for detailed temporal analysis.
- Continuous unattended operation: it was important for the devices to operate continuously over extended periods to minimize personnel interventions.
- Data accessibility in near real-time: to prevent further post-processing of the data and also to support timely analysis and internal decision-making processes (e.g., maintenance scheduling).
- Documented performance: proven performance in prior research and/or market presence was also of key consideration.

The selected products were (in parenthesis the abbreviations employed for the study to identify each system):

- AQY from Aeroqual (https://www.aeroqual.com): NO_2_, O_3_, PM_2.5_ and PM_10_;
- AQMesh (AQM) from Environmental Instruments (https://www.aqmesh.com): NO_2_, NO, O_3_, CO_2_, PM_1_, PM_2.5_ and PM_10_;
- ARIsense (Ari) from QuantAQ (https://quant-aq.com): NO, NO_2_, O_3_, CO, CO_2_, PM_1_, PM_2.5_ and PM_10_;
- Zephyr (Zep) from EarthSense (https://www.earthsense.co.uk): NO, NO_2_, O_3_, PM_1_, PM_2.5_ and PM_10_;
- PurpleAir (PA; https://www2.purpleair.com): PM_1_, PM_2.5_ and PM_10_;

For more details on the specifications and hardware of each system, please refer to Table 1.

**Table 1:** Overview of sensor hardware and measurement capabilities for the sensor systems in the Main QUANT study.

System	Sensing Hardware									Time Resol.
	NO	NO_2_	O_3_	CO	CO_2_	PM	Temp	RH	Press	
PA	—	—	—	—	—	Plant. PMS5003	Bosch BME 280			2 min
AQM	Alphas. NO-B4	Alphas. NO2-B43F	Alphas. Ox-B431	—	Alphas. IRC-A1	Environ. Instr. Ltd	Sens. SHT21		Freescale MPL115A1	1 min/ 15 min
AQY	—	Aeroqual GSE	Aeroqual GSS	—	—	Nova Fitness SDS011	NA	NA	NA	1 min
Zep	Alphas. NO-A4	Alphas. NO2-A43F	Alphas. Ox-A431	—	—	Plant. PMS5003	Sens. SHT31		Bosch BME 680	1 min
Ari	Alphas. NO-B4	Alphas. NO2-B43F	Alphas. Ox-B431	Alphas. CO-B4	Alphas. IRC-A1	Part. Plus 9301P-OEM	Sens. SHT21		Bosch BMP 180	1 min

Abbreviations: “Alphas.” for Alphasense (OEM manufacturer); “Plant.” for Plantawer (OEM manufacturer); “Environ. Instr.” for Environmental Instruments (systems manufacturer); “Sens.” for Sensirion (OEM manufacturer); “Part. Plus” for Particles Plus (OEM manufacturer); “NA” for “Not Available”.

Following more than one year after starting the Main QUANT study, the WPS phase was initiated. Offered at no cost, the call for participation in this stage was publicly announced in March 2021, leveraging the established test-bed infrastructure to demonstrate sensor performance. The WPS encompassed a wider array of platforms and was exclusively carried out at MAQS, (as detailed in “Sites selection”), with manufacturers supplying a minimum of two sensor devices each. The participating products were:

- Atmos (Atm) from Urban Sciences (http://urbansciences.in/): PM_1_, PM_2.5_ and PM_10_;
- IMB from Bosh (https://www.bosch-mobility-solutions.com): NO_2_, O_3_, PM_2.5_ and PM_10_;
- Polludrone (Poll) from Oizom (https://oizom.com): NO, NO_2_, O_3_, PM_2.5_ and PM_10_;
- Kunak Air Pro (AP) from Kunak (https://www.kunak.es/): NO, NO_2_, O_3_, CO, PM_1_, PM_2.5_ and PM_10_;
- Silax Air (SA) from Vortex (https://vortexiot.com): NO_2_, O_3_, PM_2.5_ and PM_10_;
- Node-S (NS) from Clarity (https://www.clarity.io): NO_2_, PM_1_, PM_2.5_ and PM_10_;
- Praxis/Urban (Prax) from South Coast Science (https://www.southcoastscience.com): NO, NO_2_, O_3_, CO_2_, PM_1_, PM_2.5_ and PM_10_.
- Modulair-PM (Mod) from QuantAQ: PM_1_, PM_2.5_ and PM_10_;
- AQMesh (AQM): NO_2_, NO, O_3_, CO_2_, PM_1_, PM_2.5_ and PM_10_;

Details about the measured variables and the hardware components of these devices are presented in Table 2.

**Table 2:** Overview of sensor hardware and measurement capabilities for the sensor systems in the WPS study.

System*	Sensing Hardware									Time Resol.
	NO	NO_2_	O_3_	CO	CO_2_	PM	Temp	RH	Press	
Mod	—	—	—	—	—	Alphas. OPC-N3 & Plant. PMS5003	NA	NA	NA	1 min
Atm	—	—	—	—	—	Plant. PMS7003	Adafruit DHT22		—	2 min
IMB	—	NA	NA	—	—	NA	NA	NA	NA	1 min
Poll	Alphas. NO-B4	Alphas. NO2-B43F	Alphas. Ox-B431	NA	NA	Wuhan Cubic PM3006S	NA	NA	NA	10 min
AP	Alphas. NO-B4	Alphas. NO2-B43F	Alphas. Ox-B431	Alphas. CO-B4	Alphas. IRC-A1	Alphas. OPC-N3	NA	NA	NA	5 min
SA	—	NA	NA	—	—	NA	NA	NA	NA	5 min
NS	—	Alphas. NO2-A43F	—	—	—	Plant. PMS6003	Bosch BME280			~5 min
Prax	Alphas. NO-A4	Alphas. NO2-A43F	Alphas. Ox-A431	Alphas., CO-A4	Alphas. IRC-A1	Alphas. OPC-N3	Sens. SHT31		TDK	1 min

*For AQMesh details and abbreviations see Table 1.

### Co-location sites

**Fig. 2:**
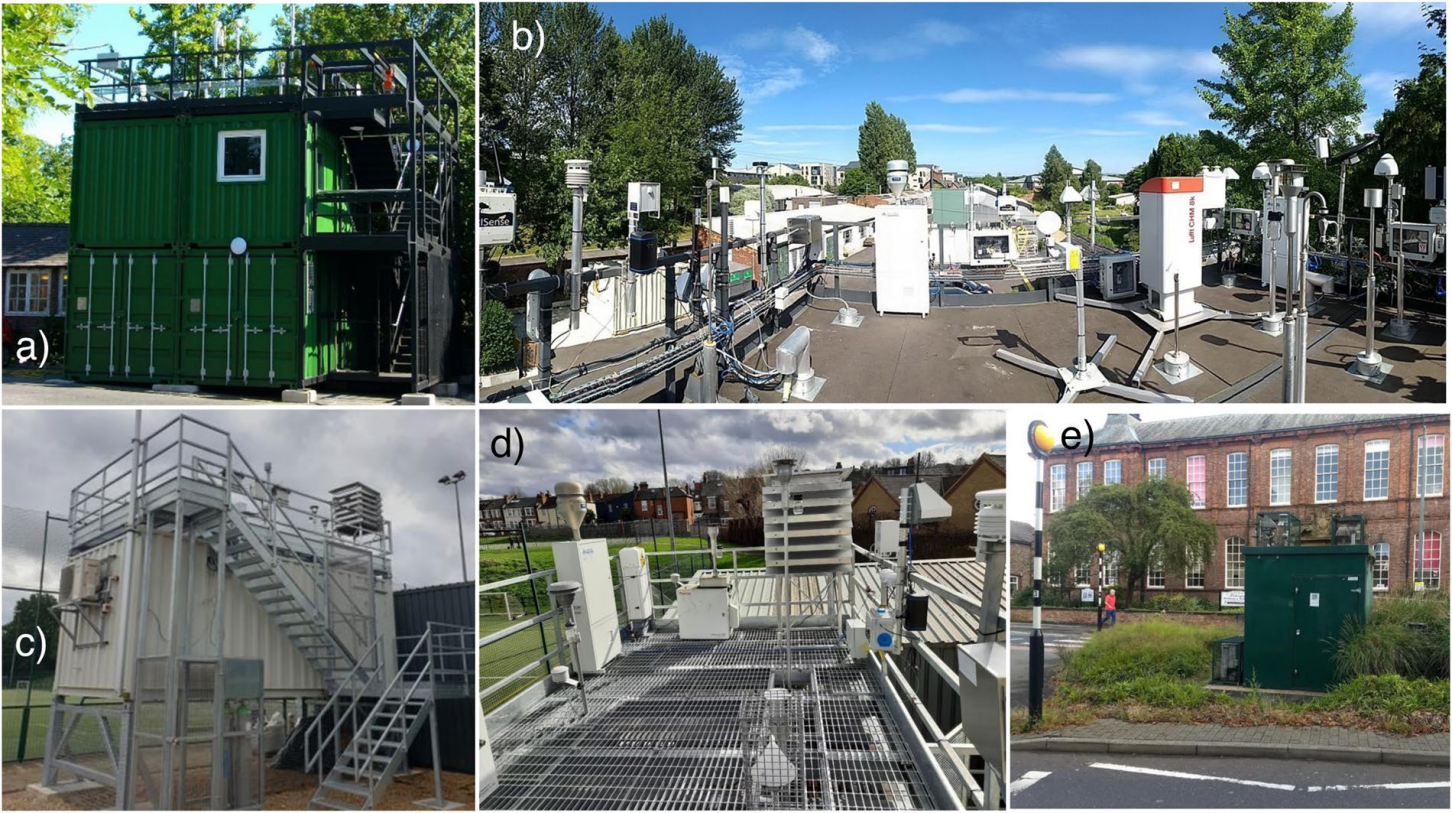
Panels (a,b) show the Manchester Air Quality Supersite (MAQS) site, characterized by its range of atmospheric monitoring instruments in an urban environment. Panel (b) provides a view of the rooftop setup with multiple sampling devices. Panels (c,d) present the London Air Quality Supersite (LAQS). Panel (c) shows a general perspective of the site, while panel (d) provides a close-up of the measurement devices installed on the roof. Finally, panel (e) illustrates the York air quality monitoring site (YoFi), located next to a road, with a single green container housing the sensor systems on the roof, and the reference instruments inside.

For the Main QUANT deployment, three different field sites across the UK were selected in order to capture a variety of conditions. This included two extensively equipped urban background supersites (i.e., MAQS and LAQS), plus a roadside monitoring site (i.e., YoFi). The selection of these sites was based on three primary criteria: (i) extensive instrumentation for measuring the chemical composition and physical properties of the atmosphere, (ii) practical considerations, such as ease of access, available space, and continuous technical assistance from site managers, and (iii) the inclusion of at least one representative roadside site. Of the three urban supersites currently available in the UK—LAQS, MAQS, and the Birmingham supersite—only two were considered due to funding constraints. MAQS was selected for its practical aspects, including ample space for installing a large number of sensors, easy access, full-time dedicated technical personnel, and transportation facilities to and from the site. London was chosen for its uniqueness in the UK, both in terms of population size and emission profile. Additionally, the space available for sensor deployment at LAQS and the on-site technical assistance made it the second site selected. The YoFi roadside site was chosen as the third site due to its ease of access and the support received from its administrators, allowing for the accommodation of additional instrumentation. Although the highly instrumented Marylebone Road site in the UK was considered, logistical and cost constraints limited its selection. Furthermore, the high traffic volume on this central London road makes it less representative of typical roadside sites across the UK, where low-cost sensors are commonly deployed. Figure 2 shows some panoramic pictures of the sites.

These selected sites offer a wide range of reference measurements, representing chemical environments typical of UK urban areas. Given time constraints, but also motivated by the MAQS capabilities, this was the only site used for the WPS study.

**Fig. 3:**
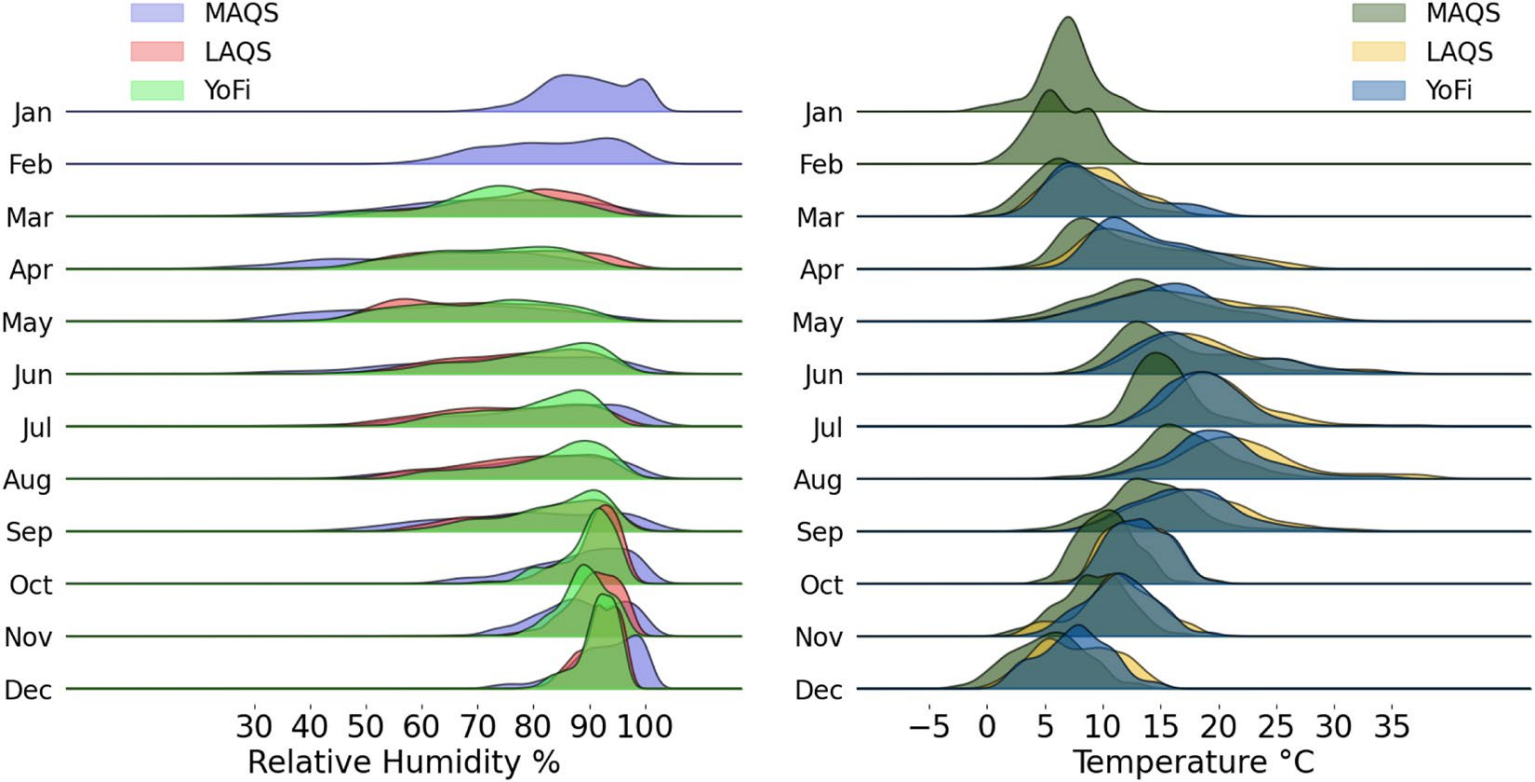
Hourly distribution of Relative Humidity (%; left panel) and Temperature (°C; right panel) for 2020 across three locations: MAQS (year-round), LAQS, and YoFi (the latter two from March onwards). These plots capture the seasonal variation of these key environmental parameters influencing sensor data quality.

MAQS measures gases, aerosols and meteorology and is one of the most extensive air quality study facilities in the UK (for detailed information visit: http://www.cas.manchester.ac.uk/restools/firs/). Located in the south of the metropolitan area (Fallowfield Campus, University of Manchester; 53° 26′ 39.2″ N, 2° 12′ 51.9″ W), it offers a typical UK urban background setting. This site is free from direct traffic emissions and surrounded by student accommodations, university buildings, and sports facilities. The neighborhood’s shops, bars, and restaurants contribute to foot traffic and vehicle movement. Additionally, emissions from heating and cooking in residential buildings affect the area’s ambient air quality. The average winter Temp at MAQS is 4–5 °C and RH is around 87%. In summer, the mean Temp is 16–17 °C with RH approximately 88% (see Fig. 3). The research-grade instrumentation used for this analysis is compounded by chemiluminescence NO analyzer (Thermo, 42i-y. Limit of detection <50 ppt, root mean square “zero” noise <25 ppt), a Cavity Attenuated Phase Shift Spectroscopy (CAPS) NO_2_ analyzer (Teledyne, T500. Limit of detection <40 ppt, root mean square “zero” noise <20 ppt), a UV photometric O_3_ analyzer (Thermo Scientific, 49i. Limit of detection <1.0 ppb, root mean square “zero” noise <0.25 ppb), and an optical aerosol spectrometer (Palas, FIDAS200. Mass range 0–10000 µg/m3, particle size range 0.18–18 µm).

LAQS (as of this writing, this site does not yet have a website) also supports the extensive measurements of gasses, aerosols and meteorology, comparable to MAQS. It is located in an urban background (Honor Oak Park; 51° 26′ 58.9″ N, 0° 02′ 14.6″ W), within the vast urban sprawl of Greater London. LAQS is surrounded by middle-class neighborhoods, parks, and green spaces, away from major roads and pollution sources. The area features low commercial activity, with local shops and restaurants barely affecting the overall noise and bustle. This setting offers a representative view of typical residential London air quality. The site experiences a winter Temp of approximately 5 °C on average and an RH of 84%, while in summer the mean Temp is around 17 °C with 72% RH. From this site, the research-grade instrumentation employed was integrated by a chemiluminescence analyzer NO analyzer (Teledyne, T200U. Limit of detection < 50 ppt, root mean square “zero” noise < 25 ppt), a CAPS NO_2_ analyzer (Teledyne, T500), a UV photometric O_3_ analyzer (Teledyne, 400E. Limit of detection < 0.6 ppb, root mean square “zero” noise < 0.3ppb), and an optical aerosol spectrometer to measure a Palas FIDAS200 for PM.

The York Fishergate (YoFi) is a roadside monitoring station embedded within a mixed-use neighborhood, very close to the York city center (53° 57′ 06.9” N, 1° 04′ 33.1” W). Located on a traffic island in a residential area, the site sits between two key lanes of Fishergate Road, close to a commercial zone with pubs and restaurants, and near Walmgate Stray’s recreational fields, blending light industrial features. This air quality monitoring station registers typical winter temperatures near 4 °C with 87% RH, and summer conditions averaging 15 °C and 80% RH. YoFi offered more diverse pollutant levels commonly associated with traffic-dominated areas, contrasting with the urban background sites like MAQS and LAQS. This site is equipped with a chemiluminescence NOx analyzer (Teledyne, T200UP. Limit of detection < 50 ppt, root mean square “zero” noise < 25 ppt) and two beta-attenuation PM monitors (Met One, BAM 1020. Mass range 0–10000 µg/m3, Limit of detection < 4.8 μg/m3 for 1-hour avg.), one dedicated to PM_2.5_ and the other to PM_10_.

Table 3 summarizes the information referring to the reference instruments for all sites.

**Table 3:** Research grade instrumentation used for the QUANT study.

Analyte	Manchester	London	York
NO	Thermo 42i-y (Chem)	Teledyne T200U (Chem)	Teledyne T200UP (Chem)
NO_2_	*Teledyne T500U (CAPS)	*Teledyne T500U (CAPS)	
O_3_	*Thermo 49i (UV)	*Teledyne 400E (UV)	—
PM	*Palas FIDAS200 (OAS)	*Palas FIDAS200 (OAS)	*Met One BAM 1020 (BA)

*Equivalent to reference.

Abbreviations and acronyms: Chem: Chemiluminescence; CAPS: Cavity Attenuated Phase Shift Spectroscopy; UV: Ultraviolet; OAS: Optical aerosol spectrometer; BA: Beta attenuation.N

### Sensor systems deployment

**Fig. 4:**
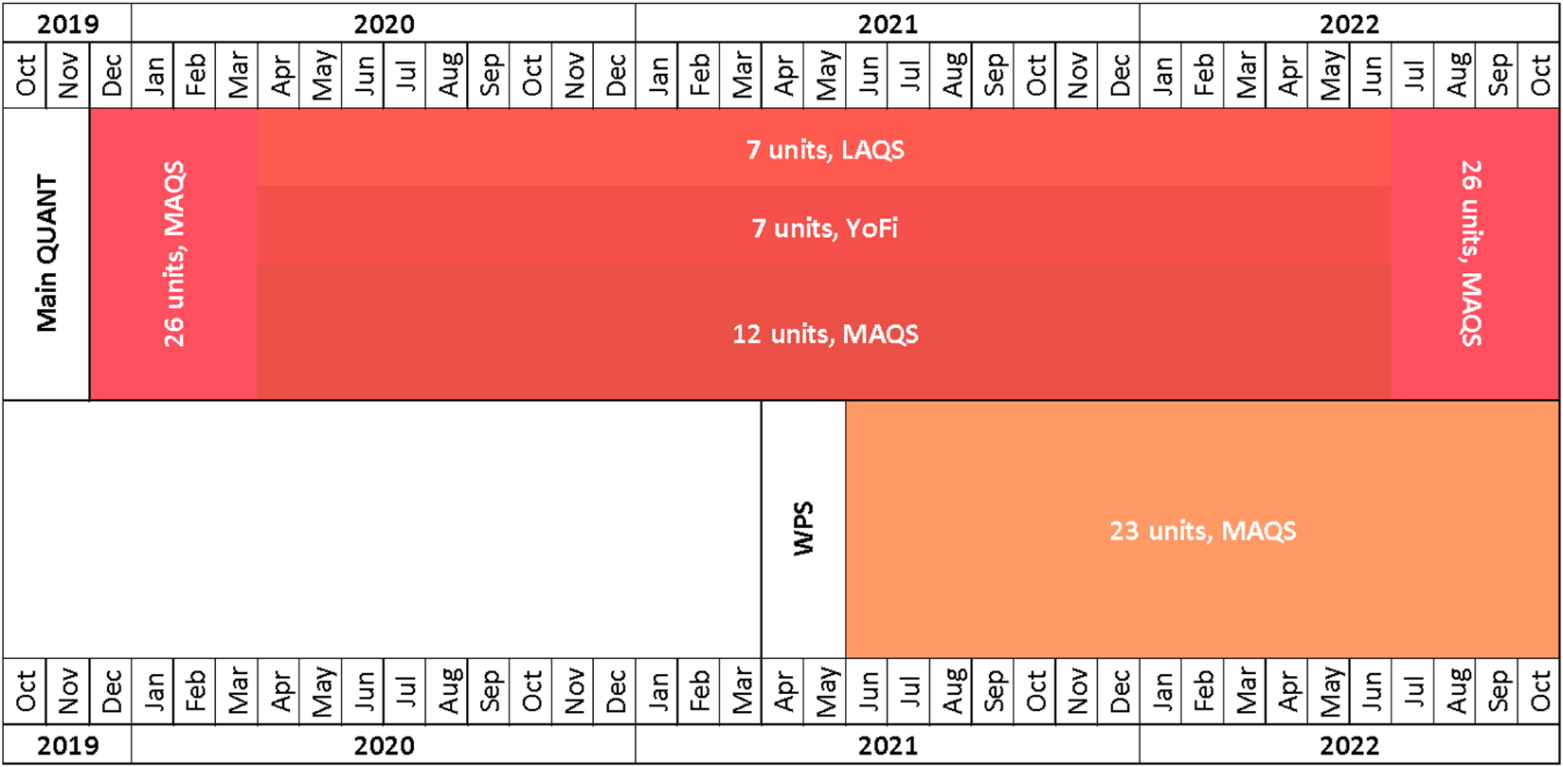
Timeline highlighting sensor deployment phases for the Main QUANT study (pinkish) from December 2019 to November 2022 across multiple UK sites (MAQS, LAQS and YoFi), and the WPS study (orange) starting June 2021 (only MAQS).

The installation of the Main QUANT systems was carried out at MAQS between December 10 and 19, 2019 (see Fig. 4). For the first three months, all systems remained at MAQS (until mid-March 2020). Subsequently, more than half of them were distributed to the other two co-location sites, LAQS (London, March 11, 2020) and YoFi (York, March 23, 2020).

For 2 years and 3 months (mid-March 2020 to early July 2022), 12 systems remained at MAQS, with the rest distributed between LAQS (7) and YoFi (7). All systems were relocated back to MAQS (July 2022) until the end of the study (November 2022). This schedule was established to initially subject all sensors to identical conditions to evaluate their performance, followed by exposure to varied environments to understand their adaptation, and finally regrouping them at MAQS to gather data reflecting the systems’ aging.

All systems were mounted on poles, acquired specifically for this project, or mounted on rails at the co-location sites. The manufacturers’ instructions were carefully followed, such as in terms of electrical installation, mounting, cleaning, and maintenance of the sensors. At YoFi, space constraints required meticulous planning to guarantee an effective co-location without compromising their operation. This involved optimizing spatial usage to maintain data integrity.

Tailored electrical setups were implemented for each sensor, considering their energy requirements. This involved using location-specific energy sources, connecting to the electrical grid with weather-resistant safety systems, and implementing security measures against vandalism. The sensors underwent maintenance checks at least once a month, except during the period of COVID-19 restrictions (March to June 2022), where site visits were limited to a maximum period of four months without on-site maintenance.

Complementary to the Main QUANT setup, the WPS was carried out exclusively at MAQS from (10 June 2021 to 31 October 2022, 16-month in total). In terms of installation and mounting, the WPS sensors were installed following the same practices as the Main QUANT, including compliance with the manufacturers’ instructions, electrical installation, mounting height and proximity to the inlets. Also, similar strategies were implemented to ensure installation efficacy and maintenance of data integrity.

### Sensor data collection

Data from all sensors were collected and processed using standardized methods throughout our study. This included maintaining uniform data logging intervals and adhering to consistent data transmission protocols (GPRS/LTE, supplemented by WiFi for specific units). To safeguard data integrity and prevent any potential data manipulation, we implemented a bespoke Extract, Transform, Load (ETL) pipeline in Python, executed daily within Amazon Web Services (AWS) containers. This automated pipeline systematically retrieved the previous day’s data from each company’s API, organizing it into a standardized CSV format. This daily retrieval ensures that data is captured and stored in near real-time, also reducing the risk of data loss or alteration. An exception involved the PurpleAir devices, which, due to connectivity challenges, required on-site data gathering and manual upload. These data were then integrated into the standardized CSV format to maintain consistency. All CSV files were securely stored in Cloud Storage (Google Drive), with strict version control and backup protocols to secure data availability and integrity.

Throughout the study, we further processed the raw data into minute-by-minute averages for consistent timestamps and batch-inserted them into a relational database (Postgres) with relevant metadata and co-located reference data using custom R and SQL scripts. No additional modifications to the original measurements were applied. The final database (outlined in the “Data Records” section) was created by converting the CSV files into NetCDF format.

### Duplicate reference instrument deployment

As part of QUANT, a specific deployment of duplicate reference instruments was conducted exclusively in Manchester. This was aimed at providing end-users with a more accurate characterization of the measurement uncertainties associated with reference methods. Initially, we planned to install duplicate monitors for PM_2.5_, NOx, and O_3_, reflecting their status as critical pollutants in UK air quality management. However, while the duplicate NOx and O_3_ monitors were successfully deployed, the installation of a second PM_2.5_ monitor encountered significant delays. These were primarily due to the COVID-19 pandemic and funding constraints, leading to its deployment only towards the end of the QUANT project. Consequently, data from this second PM_2.5_ instrument were not included in the initial dataset presented in this study.

For NOx, we utilized two Teledyne T200 instruments (employing chemiluminescence; temporal resolution ~2 min) installed in two different portions of the QUANT study. The first instrument (serial 21842), was operational from October 13, 2020 to December 17, 2020. The subsequent instrument (serial 23828), worked from March 27, 2021, to December 1, 2021.

For Ozone, two distinct devices were deployed. Initially, a 2B Technologies 202 instrument, (utilizing ultraviolet (UV) photometry; serial 312D and temp. res. of 1-2 min), was deployed from April 9, 2021, to July 29, 2021. This was complemented by a Thermo 49i instrument (also based on UV photometry; serial 1008241369 and a temp. res. ~1 min, operational from June 30, 2021 to November 19, 2021.

Quality Assurance and Quality Control (QA/QC) procedures were meticulously applied to all instruments both before and after their deployment, conducted by the skilled personnel from our lab (the Wolfson Atmospheric Chemistry Laboratories, WACL). During their co-location, these instruments adhered to the same rigorous checking routines as those already on-site, ensuring data integrity and comparability. For more detailed information on these QA/QC routines, refer to the section “Reference Data Validation”.

## Data Records

The QUANT sensors dataset is available at CEDA^24^. Within the repository, there are three folders, one per site: i.e., *fishergate*, *maqs*, and *laqs*. Refer to Table 4 for a descriptive summary. Also, in the same root directory, it can be found three files:

- *metadata.yaml*: it is a YAML format document with a detailed description of the QUANT dataset.
- *00README_catalogue_and_licence.txt*: contains information on the publication status, a link to the CEDA data catalog, and the data usage license.
- *Quant_instrument_list.csv*: it offers details about:
  - *system_id*: devices identification (internal to the project);
  - *study*: co-location studies, i.e., “QUANT” and “Wider Participation Study”.
  - *manufacturer*: company name.
  - *model*: instrument model version.
  - *url*: manufacturer’s website.
  - *serial*: manufacturer devices ID.
  - *description*: brief description of the use given to each sensor, detailing manufacturers, models, and the pollutants measured (i.e., particles, gasses, and met parameters).

**Table 4:** Distribution and count of sensor data files by brand across site folders.

*Site folder	Brands (# files)	Total number of files
*fishergate*	QuantAQ (x7)	28
	PurpleAir (x6)	
	Environmental Instruments (x6)	
	EarthSense (x5)	
	Aeroqual (x4)	
*laqs*	QuantAQ (x7)	28
	PurpleAir (x6)	
	Environmental Instruments (x6)	
	EarthSense (x5)	
	Aeroqual (x4)	
*maqs*	Environmental Instruments (x45)	211
	QuantAQ (x34)	
	PurpleAir (x20)	
	EarthSense (x20)	
	Kunak (x20),	
	Aeroqual (x16)	
	Oizom (x14)	
	South Coast Science (x12)	
	Clarity Movement (x9)	
	Vortex IoT (x9)	
	Bosch (x8)	
	Urban Sciences (x4)	

*Within each folder, there is a subfolder named *00CSV_files* which contains the same number of files with the same information but in .csv format.

It is important to note that this repository^24^ does not include reference data.

The naming of the main data files (NetCDF files) follows this convention: “*Manufacturer-system_id-variable_site_initDate-finishDate.nc*”, where:

- Manufacturer: company name;
- system_id: systems ID (internal to the project);
- variable: either a pollutant (i.e., NO2, O3, NO, PM1, PM2.5, PM10) or the meteorological (abbreviated as “Met”) variables measured by the system;
- site: identifier of the study in which the sensor was used;
- initDate: start date of the data collection by that specific sensor;
- finishDate: end date of the data collection by that sensor.

Following the naming convention detailed earlier for the data files, Table 5 provides the structure and format of the sensor system files and Table 6 outlines the quality flag variables are. For more details on the calibrated data products, refer to the “Calibrated products” section in Technical Validation.

**Table 5:** Description of the main variables and attributes of the NetCDF files.

Header	Description	Data format
*time*	- *units:* seconds since 1970-01-01 00:00:00;	int32
	- *valid_min:* minimum value;	
	- *valid_max*: maximum value;	
*sensornumber*	Corresponds to the sensing element history. If the sensor has never been replaced it’ll take a value of “1”	int16
	- *valid_min*: minimum value (equal to “1”);	
	- *valid_max:* maximum value (e.g. if it has been replaced just once it’ll take the value of 2, and so on);	
	- *date_first_measurement:* start date of measurements for a specific sensing element;	
	- *date_last_measurement:* end date of measurements for a specific sensing element;	
*calibration*	It describes the data product.	int16
	- *flag_values:* indication of the calibrated product. It can take the values of “1”, “2” or “3”;	
	- *flag_meanings*:	
	*out-of-box* (for *flag_values = *1): data product produced by default by the manufacturer;	
	*cal1* (for *flag_values = *2), first corrected/calibrated data product;	
	*cal2* (for *flag_values = *3), second corrected/calibrated product;	
	- *date_calibration_applied:* the date on which a new data product was released;	
	Note 1: NO, NO_2_, O_3_, PM_2.5_ and PM_10_ are the only variables for which suppliers generated different data products.	
	Note 2: a special case is the PurpleAir sensors, which can only take the *flag_values* “1” (*flag_meanings = atmospheric*) and “2” (*flag_meanings = indoor*) which are the default data products from that company. See^27^ for more details.	
*var (i.e., air_pressure, relative_humidity, air_temperature, pm1, pm10, pm2p5, no2, no, o3, co2, co)	Measurement values.	float64
	- *units* for meteorological variables:	
	○ *Air_pressure*: hectopascal (hPa);	
	○ *relative_humidity*: percentage (%);	
	○ *air_temperature*: Kelvin (K);	
	- *units* for the air pollutants:	
	○ *pm1, pm10 and pm2p5*: ug/m3;	
	○ *no2, no, o3, co2 and co*: ppb;	
	- *FillValue*: missing or invalid data is shown as −9999.	
	- *valid_min*: minimum valid value;	
	- *valid_max*: minimum valid value;	
	- *coordinates:* co-location sites coordinates (reference system WGS84):	
	○ LAQS: 51.449694 N, −0.037389E;	
	○ YoFi: 53.951917 N, −1.075861E;	
	○ MAQS: 53.444222 N, −2.214417E;	
	- *Ancilliary_variables*: qc_flag_*var and qc_flag_reason_*var (see Table 6 for more details).

**Table 6:** Description of the quality flag variables and attributes of the NetCDF files.

Header	Description	Data format
qc_flag_*var	Quality flag indicator.	int16
	- *flag_values*: quality indicator in integer format. It can take the values of 1, 2, 3, 4.	
	- *flag_meanings*: string format of the quality indicator. These are:	
	○ *None:* the data can be considered reliable without specific reservations;	
	○ *Info:* additional information, it does not necessarily indicate a problem.	
	○ *Warning:* data may have minor issues, but are not serious enough to invalidate the data;	
	○ *Error:* the data has significant issues that affect its reliability.	
qc_flag_reason_*var	Quality flag reason behind each flag_*var.	int16
	- *flag_values*: quality indicator in integer format. For most companies, it can take integer values between 1 and 4, but for others, this can range up to 16 (see *special cases).	
	- *flag_meanings:* for the general case, it can take the string values:	
	○ *None*: no issues;	
	○ *Retrospectively_collected:* the data wasn’t collected right after it was generated;	
	○ *Power_problem:* power outage at the site.	
	*special cases: some companies offer extra metadata (follow the links):	
	• Environmental Instruments: https://rb.gy/c1qs6t;	
	• Kunak: https://rb.gy/qoo5z3;	

### Hourly records for sensor systems, reference and duplicate reference monitors

To simplify access and enhance user interaction, we have standardized and consolidated the QUANT sensor data, reference monitor data, and duplicate reference data into a more user-friendly CSV format available on a Zenodo repository^25^. This repository contains three files:

- *QUANT_SensorSystems_hourly.csv*: it contains the complete QUANT sensors dataset in hourly averages (detailed in Table 7);

**Table 7:** Description of the variables and attributes of the “*QUANT_SensorSystems_hourly.csv*”.

Header	Description	Data format
*time*	The time format is DD/MM/YYYY HH: mm. The measurement period covered in this file extends from 10 Dec 2019 to 31 Nov 2022.	date
*location*	Sites where the measurements were taken in the UK. Alternative values:	string
	- *MAQS:* Manchester (lat 53.444222 N, long −2.214417E);	
	- LAQS: London (lat 51.449694 N, long −0.037389E);	
	- YoFi: York (lat 53.951917 N, long -1.075861E).	
*instrument*	Sensor system’s name. Alternative values for the Main QUANT study: *Ari063, Ari078, Ari086, Ari093; AQM388, AQM389, AQM390, AQM391; AQY872, AQY873, AQY874, AQY875; PA1, PA2, PA3, PA4, PA5, PA6, PA7, PA8, PA9, PA10;Zep188, Zep309, Zep311, Zep344;*	string
	Alternative values for the Wider Participation Study: *AQM1, AQM2, AQM3; AP1, AP2, AP3; Atm2, Atm1; IMB2, IMB1; Mod1, Mod2, Mod3; NS1, NS2, NS3; Poll1, Poll2; Prax1, Prax2; SA1, SA2, SA3*.	
*sensornumber*	Relates to the history of the sensing element, and can take integer values between 1 and 3. If the sensor has never been replaced, it’ll take a value of “1”.	int16
*version*	It describes the data product. Alternative values:	string
	- *out-of-box*: data product produced by default by the manufacturer;	
	- *cal1*: first corrected/calibrated data product;	
	- *cal2:* second corrected/calibrated product;.	
	A special case is the PurpleAir sensors, which can only take the alternative *atmospheric* and *indoor* which are the default data products from that company. See^27^ for more details.	
*measurand*	Short names for the air pollutants (or meteorological variables) included in the files. Alternative values are: *NO, NO2, O3, PM2.5, PM1*0*, Pressure, RelHumidity and Temperature*.	string
*measurement*	Measurement values. Units for the air pollutants: for *NO*, *NO2* and *O3*: *ppb*; for *PM*1, *PM*2*.5*, *PM10*: *ug/m*^3^; Units for meteorological variables: *Pressure:* hectopascal (hPa); *RelHumidity*: percentage (%); *Temperature*: Kelvin (K);	float64
*flag*	Quality flag indicator. Alternative values are:	string
	○ *None:* the data can be considered reliable without specific reservations;	
	○ *Info:* additional information, it does not necessarily indicate a problem.	
	○ *Warning:* data may have minor issues, but are not serious enough to invalidate the data;	
	○ *Error:* the data has significant issues that affect its reliability.	
*flagreason*	It can take integer values between 1 and 4 for most companies. For others, this can range up to 16 (see *special cases).	string
	For the general case, it can take these string values:	
	○ *None*: no issues;	
	○ *Retrospectively_collected:* the data wasn’t collected right after it was generated;	
	○ *Power_problem:* power outage at the site.	
	*special cases: some companies offer extra metadata. For comprehensive details follow the links below:	
	• Environmental Instruments: https://rb.gy/c1qs6t;	
	• Kunak: https://rb.gy/qoo5z3;
- *QUANT_Reference_hourly.csv*: it includes reference data from MAQS, LAQS, and YoFi (see Table 8 for details);

**Table 8:** Description of the variables and attributes included in the “*QUANT_Reference_hourly.csv*” file.

Header	Description	Data format
*time*	The time format is DD/MM/YYYY HH: mm. The measurement period covered in this file extends from 10 Dec 2019 to 31 Nov 2022.	date
*location*	Sites where the measurements were taken in the UK. Alternative values:	string
	- *MAQS:* Manchester (lat 53.444222 N, long -2.214417E);	
	- LAQS: London (lat 51.449694 N, long -0.037389E);	
	- YoFi: York (lat 53.951917 N, long -1.075861E).	
*versión*	Level of quality assurance of the reference data at the time it was collected. Alternative values are: *Ratified and Unratified*.	string
*measurand*	Short names for the air pollutants (or meteorological variables) included in the files. Alternative values are: *NO, NO2, O*3*, PM1, PM2.5, PM10, Pressure, RelHumidity and Temperature*.	string
*measurement*	Measurement values. The units for the air pollutants are:	float64
	- *NO*, *NO2* and *O3*: *ppb*;	
	- *PM1*, *PM2.5*, *PM10*: *ug/m*^3^;	
	and for the meteorological variables:	
	- *Pressure:* hectopascal (hPa);	
	- *RelHumidity*: percentage (%);	
	- *Temperature*: Kelvin (K);	

Note: the data included in this file contain the most up-to-date version at the time of publishing here. However, the data may have undergone further ratification processes after being published in this repository.
- *QUANT_DuplicateRef_hourly.csv*: it offers duplicate reference monitor data (refer to Table 9).

**Table 9:** Description of the variables and attributes included in the “*QUANT_DuplicateRef_hourly.csv*”.

Header	Description	Data format
*time*	The time format is DD/MM/YYYY HH: mm.	Date
	Although the file data goes between 13 Oct 2020 to 19 Nov 2021, each instrument included here covers different sub-periods:	
	- Teledyne T200 (serial 21842): 13 Oct 2020 to 17 Dec 2020;	
	- Teledyne T200 (serial 23828): 27 Mar 2021 to 27 Oct 2021;	
	- 2B 202 (serial 312DB): 9 Apr 2021 to 27 Jul 2021;	
	- Thermo 49i (serial 1008241369): 30 Jun 2021 to 19 Nov 2021;	
*location*	The site name. *MAQS* for all cases (lat 53.444222 N, long -2.214417E);	string
*instrument*	Brand and model of the instrument (i.e., *Teledyne_T200*, *2B_202* and *Thermo_49i*).	string
*serial*	Unique ID for each instrument to unequivocally identify each monitor.	string
*measurand*	Type of gas measured. Options are: *NO*, *NO2*, *O3*.	string
*measurement*	Measured values of the gasses (in parts per billion, ppb).	float64
*qc_flag*	Data quality associated with the measurements. Options are:	int16
	- *0* = good data*;*	
	- *1* = below detection limit*;*	
	- *2* = reduced quality data*;*	
	- *3* = missing data.

The choice of CSV format for the QUANT dataset improves its accessibility, leveraging its widespread familiarity to facilitate ease of use compared to the more complex NetCDF files housed at the CEDA repository^24^. These files, while robust, often challenge end-users with their volume and technical demands. Additionally, accessing reference data from MAQS, LAQS, and YoFi involves navigating multiple repositories, which are not necessarily easy to find and vary in terms of data origin, accessibility, and format (e.g., variable naming uniformity, physical units, and time formatting). Our streamlined approach enhances the dataset’s utility for diverse end-users, supporting more effective analysis.

### Interactive data visualization platform: the QUANT Shiny app

To further our commitment to data accessibility, especially for non-experts, we have developed a user-friendly platform called QUANT Shiny app (https://shiny.york.ac.uk/quant/). Currently under active development, this platform facilitates the exploration of the dataset through interactive visualizations and basic analysis. This tool is publicly accessible and allows users to select data products (O_3_, NO_2_, PM_2.5_, and various calibration versions), sensors by brand, co-location periods, and preview performance characteristics like time series for sensors and reference instruments, Bland-Altman plots, regression plots, including the regression equation, the Coefficient of Determination (R²), and the Root Mean Squared Error (RMSE). This tool enhances the dataset’s practical utility.

## Technical Validation

The overarching aim of the QUANT dataset is to provide end-users means to characterize the performance of current commercial air quality sensors and to assess the associated uncertainties across different systems and brands under real-world conditions.

### Sensor data quality assurance and quality control

To guarantee the consistency and comparability of the data collected, and to mitigate the impact of external factors on sensor performance, all sensors were deployed, maintained, and operated under identical conditions. This standardized methodology included uniform installation procedures, operational settings, QA/QC protocols, maintenance schedules, and documentation practices.

All sensors across these three sites were subjected to identical testing conditions during the co-location period. Sensors were placed within 3 meters of the reference instruments’ inlets to maximize data representativeness and accuracy, given the rapid changes in urban environments.

The measurement capabilities at each site allowed for the monitoring of critical parameters such as temperature, relative humidity, along with other potential confounders (e.g., wind speed, and interfering gasses like O_3_, CO and CO_2_) to be rigorously monitored. This enhances the reliability of the QUANT dataset, enabling end-users to accurately assess the influence of environmental factors on the observed variations in sensor performance.

Data integrity was maximized through daily retrievals, with periodic comparisons against the data available on the manufacturer’s cloud to verify that no unauthorized post-collection modifications had been made to the data. Throughout the study, no undue changes were identified.

No data post-processing aiming at improving data product quality was performed on the data from the sensor devices by the QUANT team. This was to ensure that the data collected in this study is representative of that collected by any end-user of these technologies. The data processing done by sensor device manufacturers prior to reporting of the data is treated as confidential intellectual property by the majority of device manufacturers, and as such is unknown to the QUANT team, and any other end-user.

Minor processing was carried out to prepare the data files, including aligning data to standard formats and applying time averaging where necessary. We did not apply any modifications or imputations to the original measurements. Missing values, regardless of the cause, were preserved as missing to maintain the authenticity of the dataset.

Potential issues (e.g., malfunction, disruption of data, anomalies, etc.) were closely monitored through a master record (internally called “Units Log”; see “Documentation practices” for more details) and daily summary emails sent to the QUANT team providing quantitative information for each company and sensor ID, detailing the percentage of data received (i.e., timestamps, pollutant and environmental variables measurements). It utilized a color-coded system (green for “all OK”, yellow for “attention needed”, and red for “potential issues”), offering a quick qualitative insight into the instrument’s status. This dynamic monitoring allowed us to take preventive actions, such as addressing the deterioration in data reception over time, and corrective measures, such as immediate intervention. Basic manual time series analysis was also used to identify early signs of sensor malfunctions, facilitating proactive maintenance. In cases of data disruption, the first response was to consult site administrators (MAQS, LAQS, YoFi); if unresolved, the QUANT team contacted suppliers for further support. Site visits were arranged as necessary to inspect and maintain the devices. Detailed metadata also document periods when instruments were non-operational, with reasons for these outages noted (see “Documentation practices”).

### Documentation practices

Rigorous documentation practices were implemented throughout QUANT. These centered around the “Units Log”, supplemented by the documentation provided by sensor manufacturers and the site managers. Maintained manually on a daily basis by our team, the master record was used to log day-by-day information for each site and sensor, including reference instruments from the sites and our own. It documented a range of data: installation/de-installation events, instrument locations; operational status (sensors and reference monitors), changes in operational conditions, calibrations performed; records of cartridge and unit changes, errors, failures, power outages, maintenance visits, and sensor replacements; links to internal documents (such as site audits, calibration certificates, manufacturers’ operational procedures, contracts and service agreements, software updates documentation, site plans, communication logs, technical decision records, and incident and problem resolution reports) as well as external resources like company dashboards links and relevant websites. This enabled a comprehensive oversight of sensor functionality and the identification and resolution of issues.

Relevant information collected through the Units Log was associated with corresponding metadata in the database, including software details (e.g., calibration versions), hardware information (e.g., parts replacements), and manufacturer-provided flags. This enhances the traceability of each measurement.

### Sensor data availability

**Fig. 5:**
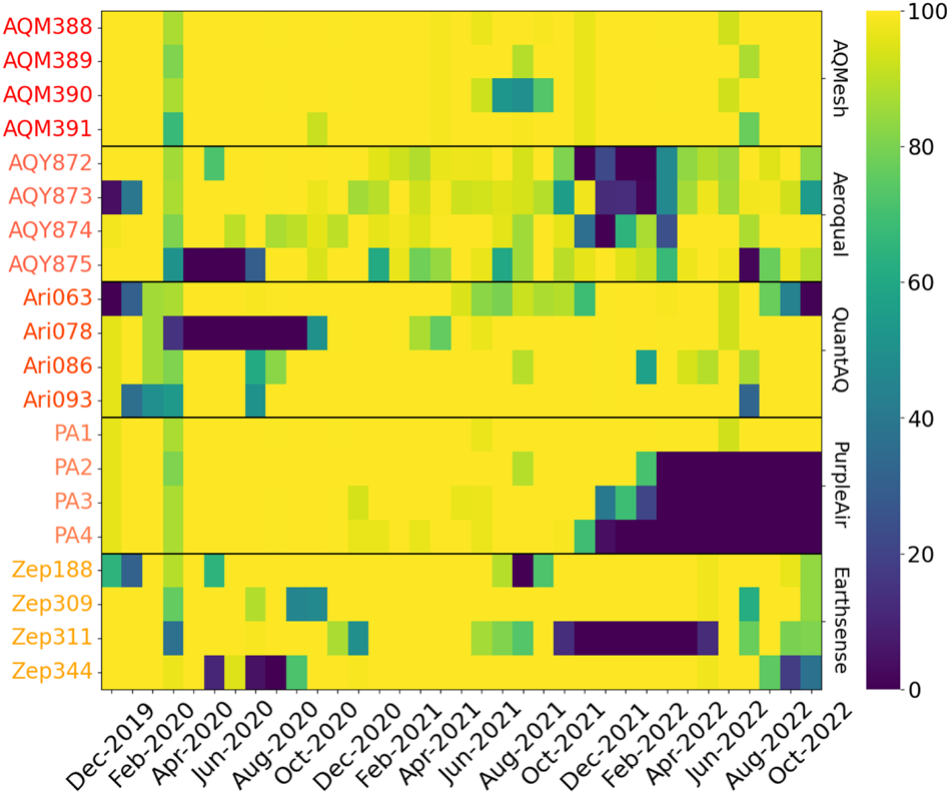
Main QUANT data availability. The colors represent the percentage of hourly data available per day per instrument (%). The black horizontal lines delimit each brand. Dark blue colors show instruments “out of service” caused by different reasons.

**Fig. 6:**
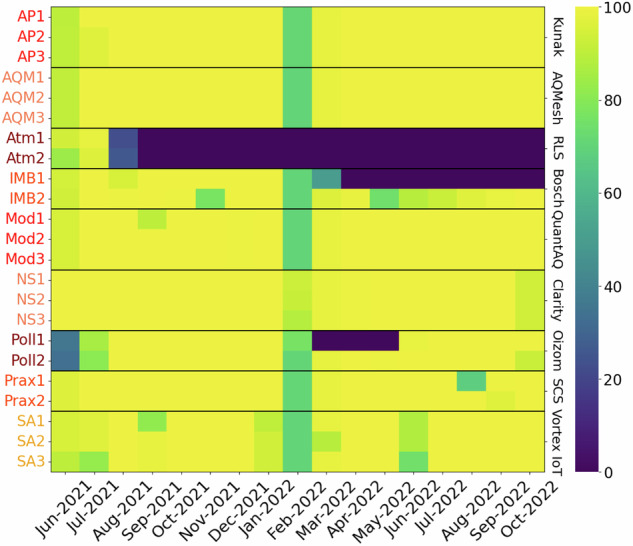
WPS data availability. The colors represent the percentage of hourly data available per day per instrument (%). Black horizontal lines delimit each brand and dark blue colors show instruments “out of service”.

During QUANT a number of device failures resulted in lost data (see Figs. 5, 6), with some of the most significant issues experienced during the Main QUANT assessment being: mechanical malfunction, connectivity, water ingress, power supply failures, and wiring/connector failures. Some specific problems include a compromised SD card causing the on-board computer to fail (e.g. AQY875, missing data from Feb to May 2020), moisture seeping into a power supply PCB due to a broken seal (e.g. Ari078, missing data from Feb to Jul 2020), a main unit chip failure compounded by supply chain delays for a replacement (e.g. Zep311, missing data from Sep 2021 to Mar 2022 & Poll1, from Feb to Apr 2022), and sudden irreversible failure (e.g. PA2, 3 and 4, after Nov 2021; Atm1 and 2 after Aug 2021).

### Reference data sharing

We periodically supplied the manufacturers with retrospective reference data on gas and particulate phase pollutants. This would allow makers to use the data for (i) validation, (ii) corrections, (iii) calibration and (iv) benchmarking of their products. In turn, suppliers were expected to provide updated data products if they developed new ones during this process.

Data sharing was conducted in three phases, each consisting of one-month periods of reference data. After the close of each data collection period, we shared preliminary reference data, allowing manufacturers for an immediate check into the sensors’ performance. Once the reference data was audited (process called “data ratification”. For more details see “Reference data validation”) by the National Physics Laboratory (NPL, UK), we distributed the ratified data. The intervals between these sharing phases were set at approximately six months, providing manufacturers with ample time to analyze and apply any correction to their devices.

To preserve the fairness and integrity of the evaluation process, all reference data was kept under embargo until it was ready to be released. Once available, it was disseminated simultaneously to all participating manufacturers. Table 10 outlines the dates and durations for each reference data release for both the Main QUANT and the WPS.

**Table 10:** Timeline for the release of the reference data during the Main QUANT and WPS.

Study phase	Period of reference data	Date released
Main QUANT	10 Dec 2019 - 17 Feb 2020	15 Apr 2020
	18 Feb - 17 Aug 2020	27 Oct 2020
	18 Aug 2020 - 17 Feb 2021	15 Apr 2021
WPS	17 Jun to 16 Jul 2021	23 Jul 2021
	1 to 31 Dec 2021	26 Jan 2022
	1 to 31 May 2022	15 Jun 2020

### Reference data validation

The procedures implemented for reference data validation at MAQS and LAQS are as follows:

- for NO, regular calibration checks are carried out at least once a month. These include zero and span checks using a calibrated standard cylinder and a scrubber to remove any trace gasses that may interfere with the measurements. Following these checks, any necessary corrections to zero and span values are applied to uphold measurement accuracy.
- for NO_2_, daily automatic zero and span checks are applied. These are facilitated by an internal NO_2_ diffusion tube and scrubber. Zero values are corrected in response to these checks, and the span readings are closely monitored for any indications of instability.
- for O_3_, zero and span corrections are daily and automatically applied using an internal O_3_ lamp and a scrubber daily. Adjustments are made to the zero readings, and span checks provide insight into the stability of the readings.
- for CO, the instrumentation is checked every three hours for zero and monthly for span with the use of an onsite standard gas cylinder. Both zero and span values are then adjusted based on these frequent checks.
- for CO_2_, stability checks are regularly performed using an onsite cylinder, although these checks do not lead to direct corrections.
- for PM, the QA/QC process involves the verification of sizing response using manufacturer-provided Mono dust, and flow rates are confirmed with a Gilibrator flow calibrator.

To warrant sustained quality and consistency, all instruments are set to continuously log operational parameters. These parameters are systematically monitored, and any deviations from the established ranges trigger automatic alerts to the site operators and the inclusion of flags within the data records.

In addition to these procedures, both sites undergo biannual data ratification audits conducted by NPL, which include comparisons with external gas standards, along with assessments of sizing and flow for PM. Any final data corrections are informed by audit results, which help define the concentration values for the onsite standards.

In the case of YoFi, the standard procedures set out in^26^ are followed. For gas analyzers (NO, NO_2_, O_3_, CO and CO_2_), routine QA/QC procedures include:

- Regular manual and automatic calibrations of analysers: zero and span controls, and stability checks, using certified calibration standards and contaminant-specific equipment.
- Site audits and network intercalibrations are carried out at semi-annual intervals by the QA/QC unit, providing a detailed assessment of network performance and compliance with national metrology standards.

For particle analyzers, similar procedures include:

- Verification of size response and flow rates using manufacturer-specific standards and calibrators.
- Semiannual zero checks to identify high baseline responses in the absence of particulate matter, with corrections applied based on the results of these tests.

### Calibrated data products

During the full QUANT study (Main QUANT and WPS), the calibration of sensor devices was conducted exclusively by the manufacturers, without any intervention from our research team. This was chosen to warrant that the sensor outputs and any subsequent calibrations mirrored the experience of standard consumers in the market. This arrangement enabled manufacturers to engage in an independent review and, if they chose, to apply this data towards the creation and submission of advanced calibrated data products. However, it’s important to note that not all manufacturers opted to incorporate this reference data for improving their calibrations. For those who did take advantage of this option, the result was a set of updated data products. These were treated as separate and distinct data versions and included various iterations such as “out-of-box” (the initial data provided with no additional calibration), “cal1” (the first round of calibrations), and “cal2” (subsequent calibration adjustments). Tables 11, 12 provide a summary of these different data products.

**Table 11:** QUANT data products and calibration-related information.

Sensor	Time resolution	Pollutants	Product (Period)		
PA	2 min	PM_1_, PM_2.5_, PM_10_	OOB (Dec 2019-Oct 2022)		
AQM*	15 min/ 1 min	CO_2_, NO, NO_2_, O_3_, PM_1_, PM_2.5_, PM_10_	OOB (Dec 2019-Jun 2020)	Cal1 (Jun 2020-Mar 2021)	Cal2 (Mar 2021-Oct 2022)
AQY	1 min	NO_2_, O_3_, PM_2.5_, PM_10_	OOB (Dec 2019-Apr 2020)	Cal1 (Mar 2021-Oct 2022)	
Zep**	1 min	NO, NO_2_, O_3_, PM_1_, PM_2.5_, PM_10_	OOB (Dec 2019-May 2020)	Cal1 (May 2020-Oct 2022)	
Ari	1 min	CO_2_, CO, NO, NO_2_, O_3_ PM_1_, PM_2.5_, PM_10_	OOB (Dec 2019-Jul 2020)	Cal1 (Jul 2020-Apr 2021)	Cal2 (Apr 2021-Oct 2022)

OOB: out-of -the-box

*AQMesh provided 15 min time res. until Feb 2020

**Zephyr only calibrated NO.

**Table 12:** WPS data products and calibration-related information.

Sensor	Time resolution	Pollutants	Product (Period)		
Mod	1 min	PM_1_, PM_2.5_, PM_10_	OOB (Jun 2021-Oct 2022)		
AQM	15 min	CO_2_, CO, NO, NO_2_, O_3_ PM_1_, PM_2.5_, PM_10_	OOB (Jun-Aug 2021)	Cal1 (Aug 2021-Mar 2022)	Cal2 (Mar-Oct 2022)
Atm*	1 hr	PM_1_, PM_2.5_, PM_10_	OOB (Jun-Aug 2021)		
IMB	1 min	NO_2_, O_3_ PM_2.5_, PM_10_	OOB (Jun-Sep 2021)	Cal1 (Sep 2021-Oct 2022)	
Poll	10 min	CO_2_, CO, NO, NO_2_, O_3_ PM_2.5_, PM_10_	OOB (Jun-Jul 2021)	Cal1 (Jul 2021-Oct 2022)	
AP	5 min	CO_2_, CO, NO, NO_2_, O_3_ PM_1_, PM_2.5_, PM_10_	OOB (Jun-Aug 2021)	Cal1 (Aug 2021-Feb 2022)	Cal2 (Feb-Oct 2022)
SA	5 min	NO_2_, O_3_ PM_2.5_, PM_10_	OOB (Jun-Nov 2021)	Cal1 (Nov 2021-Oct 2022)	
NS	~5 min	NO_2_, PM_1_, PM_2.5_, PM_10_	OOB (Jun-Aug 2021)	Cal1 (Aug 2021-Mar 2022)	Cal2 (Mar-Oct 2022)
Prax	1 min	CO_2_, NO, NO_2_, O_3_ PM_1_, PM_2.5_, PM_10_	OOB (Jun 2021-Oct 2022)		

*Catastrophic failure

OOB: out-of -the-box.

### Limitations of the QUANT dataset

The QUANT dataset, while comprehensive, is subject to several limitations that are inherent to the use of air quality sensors. The study tested a limited array of sensors and brands over a specified duration, which may not capture the diversity of technologies available. Sensor performance can vary due to environmental factors, affecting their chemical sensitivity and physical responses. Additionally, calibration procedures varied as each manufacturer applied their own standards, beyond our control potentially leading to (internal-to-the-systems) data inconsistencies. Rapid technological advancements may also date the findings, limiting their future applicability. Moreover, the specific conditions tested in the UK may not be directly extrapolatable to other regions with different atmospheric compositions and climate conditions. Users should remain cautious of these limitations when interpreting the dataset and drawing conclusions from it, particularly when applying the findings to different environmental conditions or sensor configurations not directly tested in this study.

## Usage Notes

Besides the R Shiny web app (https://shiny.york.ac.uk/quant/) described in the “Interactive data visualization platform: the QUANT Shiny app”, a repository containing Python and R code for estimating some diagnostic plots and metrics developed for QUANT can be found in this GitHub repository: https://github.com/wacl-york/quant-air-pollution-measurement-errors. It also includes examples taken from the QUANT dataset.

## Data Availability

The data retrieval pipeline was written in Python (version 3.7.6) and ran in Docker on AWS Fargate (this code is found in https://github.com/wacl-york/quant-scraper). The post-processing code to upload the data from the daily CSVs into the Postgres database (version 14.10), and then to export the database into NetCDF and CSV for storage into the CEDA repository was written in R (version 4) (found in https://github.com/wacl-york/quant-tools).
